# Multiple substance use and associated factors in transgender women and *travestis*: findings from the TransOdara Study, Brazil

**DOI:** 10.1590/1980-549720240011.supl.1

**Published:** 2024-08-19

**Authors:** Jurema Corrêa da Mota, Sandro Sperandei, Raquel Brandini De Boni, Inês Dourado, Maria Amélia de Sousa Mascena Veras, Francisco Inácio Bastos

**Affiliations:** I Fundação Oswaldo Cruz, Institute of Scientific and Technological Communication and Information in Health – Rio de Janeiro (RJ), Brazil.; II Western Sydney University, Translational Health Research Institute – Penrith (NSW), Australia.; III Universidade Federal da Bahia, Institute of Public Health – Salvador (BA), Brazil.; IV Santa Casa de São Paulo, School of Medical Sciences –São Paulo (SP), Brazil.

**Keywords:** Illicit drugs, Alcohol, Tobacco, Transgender women, Health surveys, Brazil

## Abstract

**Objective:**

To estimate the prevalence of concomitant substance consumption and analyze associated risk factors in a non-probabilistic sample of the Brazilian population of transgender women and *travestis*.

**Methods:**

A cross-sectional study was conducted with recruitment via respondent-driven sampling. The sample included transgender women and *travestis* residing in São Paulo, Porto Alegre, Salvador, Manaus, and Campo Grande, aged 18 years or older, between 2019 and 2021. The outcome was the concomitant use of licit and illicit substances. The association between sociodemographic/behavioral factors and the outcome was analyzed through Poisson regression with mixed effects. Adjusted prevalence ratios (confidence interval of 95% — 95%CI) were estimated.

**Results:**

The prevalence in the last 12 months of multiple substance use was 49.3%, of which 65.5% were alcohol, 52.9% tobacco, and 40.1% marijuana. Transgender women and *travestis* who use multiple substances face more violence (1.71; 95%CI 1.14–2.55), unemployment (1.58; 95%CI 1.05–2.37) and pervasive unstable work status (1.52; 95%CI 1.08–2.14), transactional sex (1.51; 95%CI 1.21–1.88) which can be their sole option to make a living, and are aged 18 to 24 years (1.37; 95%CI 1.14–1.65).

**Conclusion:**

The use of multiple substances may be an attempt to cope with distress and marginalization. Substance use has been associated with multiple harms and medical conditions. Comprehensive management and care should be provided, as defined by the key principles of the Brazilian Unified Health System. Health care should be integrated into structural interventions.

## INTRODUCTION

Studies on transgender women and *travestis* are still scarce worldwide, although the number has increased in recent years. This population has been overlooked by the biomedical sciences, notwithstanding seminal publications in the field of psychology^1^.

A brief search in the Medical Literature Analysis and Retrieval System Online (MEDLINE) showed that the combination “transgender health” was absent from this database until the late 1990s, and even since then, publications never exceeded a dozen indexed articles per year. It was only in the current decade that there was a substantial increase in publications on this population’s health in MEDLINE, besides the first textbook specifically dedicated to methods of studying health with gay men, lesbians, and trans persons^2^.

Even so, personal identification with a nonbinary gender is still associated with stigma, discrimination, and violence. These factors limit educational and employment opportunities and act as risk factors for various health problems such as human immunodeficiency virus (HIV) infection, mental disorders, and substance use^3,4^.

Specifically regarding substance use, the literature has shown that transgender persons present a higher prevalence of use than the general population^5^. A study conducted in Massachusetts found that 47.0% of participants reported binge drinking, 39.6% cannabis use, and 10.8% multiple substance use^6^. Factors associated with concomitant substance use feature histories of intimate partner violence, depression, posttraumatic stress disorder, discrimination, housing instability, and transactional sex^6-8^.

This involves a second, vital aspect, namely that transgender women and *travestis* are a heavily stigmatized and marginalized population that is not even acknowledged and accepted as such in some places. The research production is obviously influenced not only by the vigor and size of the respective scientific communities but also by the respective societies’ openness to addressing the issue^9^. A global perspective on the use of substances by transgender adults is provided by Connolly et al.^10^.

In the reference work *Social Research in the Digital Age* ^11^, the author explicitly mentions the difficulty in approaching taboo topics such as illicit substance use in settings that differ markedly from so-called “natural” contexts of social life and interaction.

We thus face a triple challenge here: dealing with an infrequently studied population in a country like Brazil, with a relatively modest scientific community heavily concentrated in a few centers of excellence, while addressing a complex topic (multiple substance use) surrounded by prejudices.

The risks associated with substance use include outcomes directly related to such use, such as dependence and disorders, and secondary outcomes from risk behaviors or diminished risk perception, such as acquiring/transmitting HIV and other sexually transmitted infections. The risks also include the high prevalence rates of substance use reported in the transgender population in various contexts where these consumption patterns have been analyzed^12,13^.

The current article estimates the prevalence of multiple substance use and analyzes associated risk factors in a sample of the Brazilian population of transgender women and *travestis*.

## METHODS

This was a cross-sectional study with data from the “TransOdara Project”, a study conducted from 2019 to 2021 under the coordination of the School of Medical Sciences of the São Paulo Mercy Hospital and the Institute of Collective Health of the Federal University of Bahia, Brazil. The project, carried out in five state capitals located in Brazil’s five major geographic regions, aimed to estimate the prevalence of syphilis, HIV infection, *Neisseria gonorrhoeae*, *Chlamydia trachomatis*, human papillomavirus, and hepatitis A, B, and C, as well as to understand the meanings attributed to syphilis infection among transgender women and *travestis* ^14^.

Respondent-driven sampling was the chosen methodology since this was a hard-to-reach, marginalized, and scattered population in terms of its presence in the social geography of the respective urban areas^15^. Thus, considering the inherent limitations of statistical inference in non-probabilistic samples^16^, the assumptions of classical probabilistic sampling^17^ do not apply to studies with this population.

The sample included transgender women and *travestis* residing in one of the cities participating in the study (São Paulo, Porto Alegre, Salvador, Manaus, and Campo Grande), and aged 18 years or older^14^. Participants under 18 years of age, with some identification with male gender, gender at birth as female, and those who had already participated in the research were excluded from the study^14^.

Participants recruited their acquaintances using a coupon system, and a maximum of three recruiters were allowed per participant to reduce homophily in recruitment. The researchers selected the first participants, the so-called seeds, after qualitative formative research to represent the heterogeneity of the population of transgender women and *travestis* according to demographic and socioeconomic conditions. In each city, 5–10 seeds launched the recruitment process. Each seed, and later, each participant, received three coupons to invite transgender women and *travestis* from their social contact network (referral chains). The incentives were U$ 10.00 as compensation for transportation and lost work time and U$ 10.00 for each transgender women and *travestis* recruited for the study. All completed a standard investigator-led questionnaire for sociodemographic information and sexual behavior, in a space reserved exclusively for this purpose^14^.

The minimum sample size was calculated to allow estimating with the proper precision, defining in advance^18^, the prevalence of syphilis (titer >1:8) among transgender women and *travestis* as an especially prevalent disease in this population.

### Outcome

The principal outcome was concomitant use of alcohol (How often do you drink alcohol? Never, once monthly or less, 2–4 times a month, 2–4 times a week, 4 or more times a week), tobacco (During the last 12 months, how often have you used tobacco products — cigarettes, cigars, pipes, rope tobacco? Never, once or twice, monthly, weekly, daily, or almost every day), and at least one additional substance (multiple substance use) in the 12 months before the study (During the last 12 months, how often did you use these substances — marijuana, cocaine, crack, amphetamines, inhalants, hypnotics, hallucinogens, and opioids? Never, once or twice, monthly, weekly, daily, or almost every day).

Multiple substance use was thus defined as self-reported use of one or more substances^19^, in addition to alcohol and tobacco, which are sold legally in Brazil and used by the great majority of the interviewees in this study.

### Study variables

The selected variables were age group (24 years or younger vs 25 years or older), race/skin color (white vs other), marital status (with vs without partner), studying at the time of the survey (yes vs no), schooling (primary or less vs complete secondary or greater), housing status (own home, rented, or unstable), monthly income (up to one monthly minimum wage vs greater than one monthly minimum wage), history of having suffered physical, verbal, or sexual violence or discrimination (yes vs no), self-reported health (very good or good vs fair or very bad), lifetime transactional sex (yes vs no), and screening of depression using Patient Health Questionnaire-9 (positive vs negative), with a cutoff score of 10 for the transgender population^20^.

### Statistical analysis

Prevalence ratios were calculated with the respective 95% confidence intervals (95%CI). For multivariate modeling, we applied the selection of the best set of variables that produced the Akaike Information Criterion (AIC) with the most parsimonious score.

Four regression models were fitted for this purpose and were compared to each other, drawing on the AIC, namely:

(a) model adjusted by the “backward” strategy, assisted by the *buildmer* library^21^;
(b) model with all the variables included in the study;
(c) model with the variables that proved statistically significant in the bivariate analysis; and
(d) final multivariate model that only kept the variables with significance defined by the same criteria of parsimony and fit.

All variables were tested for collinearity before the data modeling phase, considering the cutoff point of 0.60 and using the “Cramér’s V” test.

All four models used Poisson mixed regression analysis with robust variance, with random intercept referring to the city in which the interview took place, always taking the lowest risk category. Weighting was not included in the analyses, respecting the differential accuracy of the respondent-driven sampling indicators for underlying networks with varying natures^22^. The final model was the one that presented the lowest AIC. This model is addressed with the estimated prevalence ratios and respective 95%CI.

### Ethical aspects

The project was approved by the Research Ethics Committee of the Santa Casa de Misericórdia de São Paulo (CAAE 05585518.7.0000.5479; opinion n°: 3.126.815 – 30/01/2019), as well as by other participating institutions^14^.

## RESULTS

The overall prevalence of polysubstance use was found to be quite high (49.3%). The most frequently used substance in lifetime and in the previous 12 months was alcohol (65.5% in both periods). For all substances, the prevalence of use in the previous 12 months was lower than the use at any time in life. Lifetime tobacco use was 61.6%, compared to 52.9% in the previous 12 months. The rates of lifetime marijuana use and use in the previous 12 months were 52.0% and 41.0%, respectively. Lifetime cocaine use was 42.6% and in the previous 12 months, it was 31.0% (Table 1).

**Table 1:** Distribution (%) of lifetime substance use and substance use in the previous 12 months according to type of substance. São Paulo, Porto Alegre, Salvador, Manaus, and Campo Grande, Brazil, 2019–2021.

Substance	Lifetime		12 months	
	No	Yes	No	Yes
	n (%)	n (%)	n (%)	n (%)
Alcohol	437 (34.5)	828 (65.5)	437 (34.5)	828 (65.5)
Tobacco	486 (38.4)	781 (61.6)	596 (47.1)	670 (52.9)
Marijuana	608 (48.0)	659 (52.0)	759 (59.9)	508 (40.1)
Cocaine	727 (57.4)	540 (42.6)	874 (69.0)	393 (31.0)
Crack	1,091 (86.1)	176 (13.9)	1,172 (92.5)	95 (7.5)
Amphetamines/ecstasy	1,117 (88.2)	150 (11.8)	1,201 (94.8)	66 (5.2)
Inhalants	1,082 (85.4)	185 (14.6)	1,184 (93.4)	83 (6.6)
Hypnotics	1,101 (86.9)	166 (13.1)	1,165 (91.9)	102 (8.1)
Hallucinogens	1,138 (89.8)	129 (10.2)	1,202 (94.9)	65 (5.1)
Opioids	1,244 (98.2)	23 (1.8)	1,254 (99.0)	13 (1.0)
Other	1,250 (98.7)	17 (1.3)	1,257 (99.2)	10 (0.8)

Table 2 shows the distribution of sociodemographic data, participation in lifetime transactional sex, self-rated health status, victimization from violence, and the result of screening for depression, stratified by the presence of multiple substance use. Most interviewees were 25 years old or older (71.3%), and the vast majority (74.0%) reported non-white race (black, brown, indigenous, or Asian-descendent). A substantial majority were not enrolled in school at the time of the interview (78.1%), and 43.1% had completed primary schooling or less. Most interviewees lived in unstable housing conditions (74.1%), had been engaged in informal work (79.9%), and had lifetime transactional sex as their main work activity (73.7%). Half of the interviewees (50.2%) earned less than one Brazilian minimum wage per month, 91.9% had suffered some type of violence, and 43.9% showed positive screening for depression.

**Table 2:** Distribution (%) and crude prevalence ratios of multiple substance use according to sociodemographic characteristics, risk behaviors, self-rated health status, violence, and screening for depression. São Paulo, Porto Alegre, Salvador, Manaus, and Campo Grande, Brazil, 2019–2021.

Variables	Total (n=1,267)	Multiple substance use^*^		
		No 643 (50.7)	Yes 624 (49.3)	PR (95%CI)
	n (%)	n (%)	n (%)	
Sites				
Campo Grande	177 (14.0)	77 (12.0)	100 (16.0)	
Manaus	324 (25.6)	227 (35.3)	97 (15.6)	
Porto Alegre	188 (14.8)	67 (10.4)	121 (19.4)	
Salvador	191 (15.1)	91 (14.2)	100 (16.0)	
São Paulo	387 (30.5)	181 (28.1)	206 (33.0)	
Age group (years)				
18 to 24	364 (28.7)	160 (44.0)	204 (56.0)	1.27 (1.07–1.51)
25 or older	903 (71.3)	483 (53.5)	420 (46.5)	
Race/skin color				
White	328 (26.0)	167 (50.9)	161 (49.1)	1.09 (0.90–1.31)
Other	932 (74.0)	475 (51.0)	457 (49.0)	
Marital status				
With partner	182 (14.4)	94 (51.6)	88 (48.4)	
Without partner	1,083 (85.6)	549 (50.7)	534 (49.3)	1.05 (0.84–1.32)
Studying at time of survey				
Yes	277 (21.9)	135 (48.7)	142 (51.3)	0.96 (0.80–1.16)
No	988 (78.1)	507 (51.3)	481 (48.7)	
Schooling				
Primary or less	544 (43.1)	241 (44.3)	303 (55.7)	1.21 (1.04–1.42]
Secondary or more	722 (57.0)	401 (55.5)	321 (44.5)	
Housing				
Own	328 (25.9)	176 (53.7)	152 (46.3)	
Rented	465 (36.7)	238 (51.2)	227 (48.8)	1.08 (0.88–1.32)
Temporary	474 (37.4)	229 (48.3)	245 (51.7)	1.28 (1.04–1.58)
Work				
Regular	122 (9.7)	81 (66.4)	41 (33.6)	
Unstable	1,008 (79.9)	503 (49.9)	505 (50.1)	1.58 (1.14–2.18)
Not working	132 (10.5)	56 (42.4)	76 (57.6)	1.68 (1.14–2.46)
Monthly income				
BRL 0.0–BRL 1,038.00	564 (50.2)	267 (47.3)	297 (52.7)	1.13 (0.96–1.34)
≥BRL 1,038.00	559 (49.8)	285 (51.0)	274 (49.0)	
Lifetime transactional sex				
No	333 (26.3)	218 (65.5)	115 (34.5)	
Yes	931 (73.7)	423 (45.4)	508 (54.6)	1.56 (1.27–1.91)
Self-rated health status				
Very good/good	634 (50.8)	357 (56.3)	277 (43.7)	
Fair/bad/very bad	613 (49.2)	277 (45.2)	336 (54.8)	1.20 (1.02–1.41)
Violence in the previous year^†^				
No	103 (8.1)	74 (71.8)	29 (28.2)	
Yes	1,162 (91.9)	568 (48.9)	594 (51.1)	1.79 (1.23–2.60)
Screening for depression				
Positive	548 (43.9)	245 (44.7)	303 (55.3)	1.17 (1.00–1.38)
Negative	700 (56.1)	387 (55.3)	313 (44.7)	

*multiple use of alcohol AND tobacco + 1 substance (marijuana, cocaine, crack, amphetamines, inhalants, hallucinogens, sedatives, or opioids);

^†^suffered at least 1 type of violence: discrimination, verbal aggression, physical aggression, or sexual aggression. PR: prevalence ratio; CI: confidence interval.

The sociodemographic characteristics highlighted the 18-to-24-year-old group, the most common among multiple substance users (56.0%), with 1.27 times higher odds of use (95%CI 1.07–1.51) compared to subjects 25 years or older. Individuals with up to primary schooling (55.7%) had 1.21 higher odds (95%CI 1.04–1.42) of engaging in multiple substance use. Temporary housing was associated with multiple substance use (51.7%), compared to non-use (1.28; 95%CI 1.04–1.58).

Sporadic work and no work were more frequent among transgender women and *travestis* with multiple substance use (50.1%; 1.58; 95%CI 1.14–2.18 and 57.6%; 1.68; 95%CI 1.14–2.46, respectively). Participation in lifetime transactional sex was associated with multiple substance use (54.6%; 1.56; 95%CI 1.27–1.91), as were worse self-rated health (54.8%; 1.20; 95%CI 1.02–1.41), report of situations of violence (51.1%; 1.79; 95%CI 1.23–2.60), and positive screening for depression (55.3%; 1.17; 95%CI 1.00–1.38).

Table 3 shows the final adjusted model according to the lowest AIC, where the variables associated with concomitant use of alcohol, tobacco, and at least one additional substance were: age group 18 to 24 years (1.37; 95%CI 1.14–1.65), unstable work status (1.52; 95%CI 1.08–2.14) or not working (1.58; 95%CI 1.05–2.37), lifetime transactional sex (1.51; 95%CI 1.21–1.88), and exposure to some violence in the year prior to the study (1.71; 95%CI 1.14–2.55).

**Table 3:** Multivariate analysis of factors associated with multiple substance use. São Paulo, Porto Alegre, Salvador, Manaus, and Campo Grande, Brazil, 2019–2021.

Variables	aPR (95%CI)
Age group (years)	
18 to 24	1.37 (1.14–1.65)
25 or older	1.00
Work	
Regular	1.00
Unstable	1.52 (1.08–2.14)
Not working	1.58 (1.05–2.37)
Lifetime transactional sex	
No	1.00
Yes	1.51 (1.21–1.88)
Violence in the previous year^*^	
No	1.00
Yes	1.71 (1.14–2.55)

*suffered at least 1 type of violence: discrimination, verbal aggression, physical aggression, or sexual aggression. Initial model with variables that were significant in the bivariate phase AIC 1787.7; BIC 1842.5; Deviance 1765.7. Final model AIC 1785.8; BIC 1820.7; Deviance 1771.8. aPR: adjusted prevalence ratio; CI: confidence interval.

## DISCUSSION

In this study, conducted in five Brazilian state capitals, which included 1,317 transgender women and *travestis*, the prevalence of multiple substance use was 49.0% and factors associated with multiple substance use were young age, unstable work or not working, transactional sex, and having suffered some form of violence.

The target population, in which mental health disorders are common^23^, with a high prevalence of suicidal ideation^24^, also proved subject to a pattern of especially intense and diversified use of various psychoactive substances.

It would be interesting to develop more in-depth analyses of the risks and harms associated with the simultaneous use of alcohol and cocaine, extensively documented in the current sample. The two substances are metabolized concurrently by the liver, resulting in the production of cocaethylene, a highly toxic metabolite^25^. Such studies would undoubtedly require integration between the overall objective of a behavioral and serological survey and toxicology tests, which were not implemented in the current study’s context.

Another extremely relevant example involves the secondary harms and risks from the combined use of cigarettes and other smoked, inhaled, or vaporized substances (*vaping*), such as crack and electronic cigarettes. These smoked and vaporized substances display synergically adverse effects on the respiratory and circulatory systems and on the overall health of multiple substance users^26^. Since such practices are not especially frequent and do not necessarily coincide, the production of greater details would require a sample with this specific objective, providing estimates with the necessary precision to explore the phenomenon in detail.

The concentration of higher rates of multiple substance use in the specific subgroup of young individuals raises a warning signal, but also a sign of hope. This pattern of initiation of drug use appears in widely varying contexts and populations, calling attention to the need to intervene preventively and therapeutically as early as possible^27^, combining general and targeted approaches. One key aspect is to use validated screening instruments that might offer clues for health professionals and the patients themselves as part of a comprehensive routine of management and care. Once early signs become evident, each patient should be further thoroughly evaluated, and counseling and brief interventions should be offered as a first approach^28^.

Multiple drug users displayed higher odds of not being engaged in formal work, related in turn to low schooling according to a complex pattern of correlation with substance use (since intense and dependent use is often associated with increased school dropout rates). Findings from a study carried out in India, where dropout rates are of great concern, pointed to similar factors, highlighting the role of discriminatory practices and substance use^29^. Obviously, the comparison of studies from different cultures using distinct methodologies and instruments requires a cautious approach. In a cross-sectional study that did not address the issue of work and schooling in detail (since it was not the objective), it was not possible to establish the direction of association or a possible causal association between these various factors.

Education provides individuals with the resources and skills to cope better with life’s various challenges. The prevention of harmful substance use is never undertaken in a void but as part of a context of skills, knowledge, and successful coping strategies. It is no coincidence that the most successful prevention programs worldwide are not focused exclusively on substance use per se but on a set of skills and capacities^30^.

The scarce opportunities to participate in the work market mean that many transgender women and *travestis* turn to transactional sex as one of the few (or even only) possibilities to obtain income and a means of living. Transactional sex in various settings is closely associated with the consumption of substances for widely varying reasons. One example is the owners of establishments that encourage customers to consume alcoholic beverages and other substances to boost the business’s revenue and the juxtaposition of sex trade networks and sales. Besides, the use of substances is a means to cope with the work, which is frequently threatening and violent (thereby redoubling the stigma)^31^.

Beyond any dimension that affects each transgender women and *travestis* and her social networks, an overall context of transphobia, intolerance, and violence certainly contributes to the perpetuation of a vicious circle of vulnerability and low self-esteem. The harmful and dependent use of various substances is an integral part of this spiral of problems and suffering^11^ that can only be mitigated by recognition of the group’s diversity, the promotion of a culture of tolerance, and the elimination of all forms of violence.

Multicenter observational studies are thus essential, since they avoid various methodological biases, even though they create others (as mentioned), in the trade-off that characterizes common dilemmas in science.

Two of the most relevant preventable methodological biases in clinical studies involving this population group and other minorities are self-selection and avoidance. In the first case, patient self-selection, healthier individuals and those who are more concerned about their own health tend to demand more clinical care, such as patients with alcohol dependence with or without previous experience in Alcoholics Anonymous groups^32^. Closely associated with and complementing the former, are the patients known to have illegal habits with the tendency to avoid interacting with any formal services that require enrolment and regular attendance. The combination of self-selection and avoidance understandably means that a substantial share of substance users with more severe use simply fail to interact with any services and end up being identified exclusively through strategies that do not require regular human interaction of any kind, such as syringe vending machines^33^.

Referring again to the accurate analysis by Salganik, an additional bias usually known as “social desirability bias”^11^ has a strong impact not only on studies centered in clinical units but also on any studies offered to individuals that are seen (or that see themselves) as behaving according to the expected norms of a “well-behaved” and “compliant” patient. The perception is not without basis, since countless intervention protocols have rigid exclusion criteria, for example, excluding patients at risk of becoming pregnant and individuals from several minority groups, regardless of whether such exclusions are relevant^34^.

Therefore, the current set of studies belongs to a protocol ideally designed to observe a series of behaviors, attitudes, and practices that would be influenced unavoidably by a clinical protocol or biomedical intervention. Although the original protocol’s main objective was not to observe the patterns of substance use and their possible determinants, its implementation proved quite appropriate for this purpose.

A key limitation of the study refers to statistical inference to the use of non-probability samples. Recent contributions aim to improve inference, such as the use of pseudo-weights, without consensus on the use of such different methods in respondent-driven sampling studies. Different chain-referral methods have been designed to explore the basic properties of social networks, evaluating phenomena defined as simple contagions, where recruitment chains and the diffusion of a given pathogen (e.g., HIV) follow rough similar dynamics. However, this is not the case of complex behaviors^35^, and new methods of the recruitment of hard-to-reach populations with the in-depth assessment of polysubstance use have yet to be implemented^11,35^. A second limitation refers to the absence of instruments developed for the assessment of multiple substance use and misuse for the population of transgender women and *travestis*. A better understanding of the diffusion patterns of such harmful behaviors among different populations remains elusive and intervention protocols have yet to be designed and evaluated.

The Brazilian Unified Health System and the private health sector should incorporate protocols and procedures addressing the needs of different minorities, following the basic principles of comprehensiveness and integrality of care and management. This approach should not be limited to specialized care but extended to primary and community-based health care at least in its essential basics. Complex cases should be comprehensively screened and referred to specialized services, but most patients would be best managed and cared for at the community level.

Health professionals must be aware and properly trained to handle medical conditions among members of different minority groups, including gender and sexual minorities as well as people who use multiple substances. As a guiding principle, judgmental attitudes and prejudices should be avoided.

We live in a society in a fast-paced transition. Health professionals must be fully aware of the key dimension of ethics and the pressing need to provide humane treatment to patients individually, whatever his/her/their sexual and gender identities and their specific habits, including the use of licit and illicit substances.

## Supplementary Material

ONLINE SUPPLEMENT
