# Supplementary material for: Multiple substance use and associated factors in transgender women and travestis: findings from the TransOdara Study, Brazil
Source: Rev Bras Epidemiol. 2024 Aug 19;27(Suppl 1):e240011.supl.1. doi: 10.1590/1980-549720240011.supl.1 (PMC11478070; doi:10.1590/1980-549720240011.supl.1)
Supplement: Supplementary file 1 [file 1980-5497-rbepid-27-suppl1-e240011-Material-suplementar.pdf]

ONLINE SUPPLEMENT

Multiple substance use and factors in trans women: findings from the TransOdara Study, Brazil

Table S1. Comparison of the models tested to obtain the final model according to AIC, BIC, and deviance.

Variables	Model			
	**Automatic selection[a]**	**All the variables[b]**	**Significant variables[c]**	**Final[d]**
Age group: 18 to 24 versus 25 years or older	X	X	X	X
Race: white versus other		X		
Marital status: with versus without partner		X		
Currently studying: yes versus no		X		
Schooling: primary or less versus secondary or more		X	X	
Monthly income: BRL 0.00 – BRL 1,038.00 versus ≥ BRL 1,038.00		X		
Housing: own versus rented versus temporary		X	X	
Work: regular versus unstable versus not working	X	X	X	X
Screening for depression: positive versus negative		X	X	
Violence: yes versus no	X	X	X	X
Self-rated health: very good/good versus fair/bad/very bad		X	X	
Transacional sex: yes versus no	X	X	X	X
**Goodness of fit**				
AIC	1785.1	1795.7	1787.7	1785.8
BIC	1829.9	1875.5	1842.5	1820.7
Deviance	1767.1	1763.7	1765.7	1771.8

Notes:

(a) Model based on automatic selection of variables to comprise the model with the best goodness of fit

(b) Model with all the independent variables, regardless of statistical significance

(c) Initial model with the variables that showed statistical significance in the bivariate phase

(d) Final multivariate model, maintaining the variables with statistical significance
