# Supplementary material for: Multiple substance use and associated factors in transgender women and travestis: findings from the TransOdara Study, Brazil
Source: Rev Bras Epidemiol. 2024 Aug 19;27(Suppl 1):e240011.supl.1. doi: 10.1590/1980-549720240011.supl.1 (PMC11478070; doi:10.1590/1980-549720240011.supl.1)
Supplement: Translation

# Translation: Uso de múltiplas substâncias e fatores associados em mulheres trans e travestis: resultados do estudo TransOdara, Brasil

**DOI:** 10.1590/1980-549720240011.supl.1.2

**Authors:** Jurema Corrêa da Mota, Sandro Sperandei, Raquel Brandini De Boni, Inês Dourado, Maria Amélia de Sousa Mascena Veras, Francisco Inácio Bastos

**Affiliations:** I Fundação Oswaldo Cruz, Intituto de Comunicação e Informação Científica e Tecnologia em Saúde – Rio de Janeiro (RJ), Brasil.; II Western Sydney University, Translational Health Research Institute – Penrith (NSW), Austrália.; III Universidade Federal da Bahia, Instituto de Saúde Coletiva – Salvador (BA), Brasil.; IV Santa Casa de São Paulo, Faculdade de Ciências Médicas – São Paulo (SP), Brasil.

**Keywords:** Drogas ilícitas, Álcool, Tabaco, Mulheres transexuais, Inquéritos de saúde, Brasil

## Abstract

**Objetivo:**

Estimar a prevalência do consumo concomitante de substâncias e analisar fatores de risco associados em uma amostra não probabilística da população brasileira de mulheres trans e travestis.

**Métodos:**

Estudo transversal, com recrutamento por meio da metodologia *respondent-driven sampling*. A amostra incluiu mulheres trans e travestis residentes em São Paulo, Porto Alegre, Salvador, Manaus e Campo Grande, maiores de 18 anos, entre 2019 e 2021. O desfecho foi o uso concomitante de substâncias lícitas e ilícitas. A associação entre fatores sociodemográficos/comportamentais e o desfecho foi analisada com regressão de Poisson com efeitos mistos, estimando-se razões de prevalência ajustadas (intervalo de confiança de 95% – IC95%).

**Resultados:**

A prevalência nos últimos 12 meses de uso de múltiplas substâncias foi de 49,3%, sendo 65,5% álcool, 52,9% tabaco e 40,1% maconha. Mulheres trans e travestis que usam múltiplas substâncias enfrentam mais violência (1,71; IC95% 1,14–2,55), desemprego (1,58; IC95% 1,05–2,37) e trabalho instável (1,52; IC95% 1,08–2,14), sexo transacional (1,51; IC95% 1,21–1,88), que pode ser a única opção de sustento, e têm de 18 a 24 anos (1,37; IC95% 1,14–1,65).

**Conclusão:**

O uso de múltiplas substâncias pode ser uma tentativa de lidar com o sofrimento e a marginalização. O uso de substâncias tem sido associado a múltiplos danos e condições médicas. Uma gestão integral e cuidados abrangentes devem ser providenciados, conforme definido pelos princípios-chave do Sistema Único de Saúde do Brasil. Os cuidados de saúde devem ser integrados em intervenções estruturais.

## INTRODUÇÃO

Estudos sobre mulheres trans e travestis ainda são escassos em todo o mundo, embora o número tenha aumentado nos últimos anos. Essa população tem sido negligenciada pelas ciências biomédicas, apesar de publicações influentes na área da psicologia^1^.

De acordo com uma breve pesquisa no *Medical Literature Analysis and Retrieval System Online* (MEDLINE), a combinação “saúde transgênero” esteve ausente desse banco de dados até o final dos anos 1990 e, desde então, as publicações nunca ultrapassaram uma dúzia de artigos indexados por ano. Foi somente na década atual que houve um aumento substancial das publicações sobre a saúde dessa população no MEDLINE, além do primeiro livro didático dedicado especificamente aos métodos de estudo da saúde de gays, lésbicas e pessoas trans^2^.

Contudo, a identificação pessoal com um gênero não binário ainda está associada ao estigma, à discriminação e à violência. Esses fatores limitam as oportunidades de educação e emprego e são fatores de risco para vários problemas de saúde, como infecção pelo vírus da imunodeficiência humana (HIV), transtornos mentais e o uso de substâncias^3,4^.

Especificamente em relação ao uso de substâncias, a literatura tem mostrado que pessoas trans apresentam maior prevalência de uso do que a população geral^5^. Autores de um estudo realizado em Massachusetts, EUA, descobriram que 47,0% dos participantes relataram consumo excessivo de álcool; 39,6% de uso de cannabis; e 10,8% de uso de múltiplas substâncias^6^. Os fatores associados ao uso concomitante de substâncias englobam histórico de violência entre parceiros íntimos, depressão, transtorno de estresse pós-traumático, discriminação, instabilidade habitacional e sexo transacional^6-8^.

Isso envolve um segundo aspecto vital: que as mulheres trans e travestis são uma população fortemente estigmatizada e marginalizada que nem mesmo é reconhecida e aceita como tal em alguns lugares. A produção de pesquisa é obviamente influenciada não apenas pelo vigor e tamanho das respectivas comunidades científicas, mas também pela abertura das respectivas sociedades em abordar essa questão^9^. Connolly et al. providenciaram uma perspectiva global sobre o uso de substâncias por adultos trans^10^.

Na obra de referência *Social Research in the Digital Age* ^11^, o autor menciona explicitamente a dificuldade em abordar tópicos tabus, como o uso de substâncias ilícitas, em ambientes que diferem marcadamente dos chamados contextos “naturais” da vida social e da interação.

Portanto, nós enfrentamos um triplo desafio neste estudo: lidar com uma população pouco estudada em um país como o Brasil, com uma comunidade científica relativamente modesta, fortemente concentrada em alguns centros de excelência, ao mesmo tempo em que abordamos um tópico complexo (uso de múltiplas substâncias) cercado de preconceitos.

Os riscos associados ao uso de substâncias incluem desfechos diretamente relacionados a esse uso, como dependência e transtornos, e desfechos secundários de comportamentos de risco ou diminuição da percepção de risco, como adquirir/transmitir HIV e outras infecções sexualmente transmissíveis. Os riscos também incluem as altas taxas de prevalência de uso de substâncias relatadas na população trans em vários contextos nos quais esses padrões de consumo foram analisados^12,13^.

Neste artigo nós estimamos a prevalência do uso de múltiplas substâncias e analisamos os fatores de risco associados em uma amostra da população brasileira de mulheres trans e travestis.

## MÉTODOS

Trata-se de um estudo transversal com dados do “Projeto TransOdara”, um estudo realizado de 2019 a 2021 sob a coordenação da Faculdade de Ciências Médicas do Hospital da Santa Casa de Misericórdia de São Paulo e do Instituto de Saúde Coletiva da Universidade Federal da Bahia, Brasil. O projeto, realizado em cinco capitais localizadas nas cinco principais regiões geográficas do Brasil, teve como objetivo estimar a prevalência de sífilis, infecção por HIV, *Neisseria gonorrhoeae*, *Chlamydia trachomatis*, papilomavírus humano e hepatites A, B e C, bem como compreender os significados atribuídos à infecção por sífilis entre mulheres trans e travestis^14^.

A *Respondent-driven sampling* foi a metodologia escolhida, considerando que a população do estudo era de difícil acesso, marginalizada e dispersa quanto à sua presença na geografia social das respectivas áreas urbanas^15^. Assim, considerando as limitações inerentes à inferência estatística em amostras não probabilísticas^16^, os pressupostos da amostragem probabilística clássica^17^ não se aplicam aos estudos com essa população.

A amostra incluiu mulheres trans e travestis residentes em uma das cidades participantes do estudo (São Paulo, Porto Alegre, Salvador, Manaus e Campo Grande) e com idade entre 18 anos ou mais^14^. As participantes menores de 18 anos, que se identificavam de alguma forma com o sexo masculino, com o gênero feminino ao nascer e indivíduos que já haviam participado da pesquisa foram excluídos do estudo^14^.

As participantes recrutaram seus conhecidos usando um sistema de cupons e um máximo de três recrutadores foram autorizados por participante para reduzir a homofilia no recrutamento. Os pesquisadores selecionaram as primeiras participantes, chamadas de sementes, após uma pesquisa formativa qualitativa para representar a heterogeneidade da população de mulheres trans e travestis de acordo com as condições demográficas e socioeconômicas. Em cada cidade, de 5 a 10 sementes iniciaram o processo de recrutamento. Cada semente e, posteriormente, cada participante, recebeu três cupons para convidar mulheres trans e travestis de sua rede de contato social (cadeias de referência). Os incentivos foram equivalentes a USD 10,00 (na época do estudo) como compensação pelo transporte e perda de tempo de trabalho e USD 10,00 para cada mulheres trans e travestis recrutada para o estudo. Todas preencheram um questionário padrão formulado pelos investigadores para coletar informações sociodemográficas e de comportamento sexual, em um espaço reservado exclusivamente para esse fim^14^.

O tamanho mínimo da amostra foi calculado para permitir a estimativa com a precisão adequada, definindo antecipadamente^18^ a prevalência de sífilis (título > 1:8) entre mulheres trans e travestis como uma doença particularmente prevalente nessa população.

### Desfecho

O principal desfecho foi o uso concomitante de álcool (Com que frequência você consome álcool? Nunca, uma vez por mês ou menos; 2 a 4 vezes por mês; 2 a 4 vezes por semana; 4 vezes ou mais por semana), tabaco (Nos últimos 12 meses, com que frequência você usou produtos de tabaco — cigarros, charutos, cachimbos, tabaco em corda? Nunca, uma ou duas vezes, mensalmente, semanalmente, diariamente ou quase todos os dias) e pelo menos uma substância adicional (uso de múltiplas substâncias) nos 12 meses anteriores ao estudo (Nos últimos 12 meses, com que frequência você usou estas substâncias — maconha, cocaína, crack, anfetaminas, inalantes, hipnóticos, alucinógenos e opioides? Nunca, uma ou duas vezes, mensalmente, semanalmente, diariamente ou quase todos os dias).

Dessa forma, o uso de múltiplas substâncias foi definido como o uso autorreferido de uma ou mais substâncias^19^, além do álcool e do tabaco, que são comercializados legalmente no Brasil e usados pela grande maioria dos entrevistados neste estudo.

### Variáveis do estudo

As variáveis selecionadas foram faixa etária (24 anos ou menos vs 25 anos ou mais), raça/cor da pele (branca vs outra), estado civil (com parceiro(a) vs sem parceiro(a)), estudo no momento da pesquisa (sim vs não), escolaridade (ensino fundamental ou menos vs ensino médio completo ou mais), situação de moradia (casa própria, alugada ou instável), renda mensal (até um salário mínimo mensal vs mais que um salário mínimo mensal), histórico de violência ou discriminação física, verbal ou sexual (sim vs não), autoavaliação do estado de saúde (muito bom ou bom vs regular ou muito ruim), sexo transacional ao longo da vida (sim vs não) e rastreamento da depressão usando o *Patient Health Questionnaire-9* [Questionário de Saúde do Paciente-9] (positivo vs negativo), com uma pontuação de corte de 10 para a população trans^20^.

### Análise estatística

As razões de prevalência foram calculadas com os respectivos intervalos de confiança de 95% (IC95%). Para modelagem multivariada, a seleção do melhor conjunto de variáveis que produziu o Critério de Informação de Akaike (AIC), com a pontuação mais parcimoniosa, foi aplicada.

Para tal, quatro modelos de regressão foram ajustados e comparados entre si com base no AIC, a saber:

(a) modelo ajustado pela estratégia “backward”, auxiliado pelo pacote *buildmer* ^21^;
(b) modelo com todas as variáveis incluídas no estudo;
(c) modelo com as variáveis que se mostraram estatisticamente significativas na análise bivariada; e
(d) modelo multivariado final que manteve apenas as variáveis com significância definidas pelos mesmos critérios de parcimônia e ajuste.

Todas as variáveis foram testadas quanto à colinearidade antes da fase de modelagem dos dados, considerando o ponto de corte de 0,60 e usando o teste “V de Cramér”.

Os quatro modelos utilizaram análise de regressão mista de Poisson com variância robusta, com interceptação aleatória referente à cidade em que a entrevista foi realizada, sempre utilizando a categoria de menor risco. A ponderação não foi incluída nas análises, respeitando a precisão diferencial dos indicadores da metodologia *respondent-driven sampling* para redes subjacentes com naturezas variadas^22^. O modelo final foi o que apresentou o menor AIC. Esse modelo é abordado com as razões de prevalência estimadas e respectivos IC95%.

### Aspectos éticos

O projeto foi aprovado pelo Comitê de Ética em Pesquisa (CEP) da Santa Casa de Misericórdia de São Paulo (CAAE 05585518.7.0000.5479 - n^°^ parecer: 3.126.815 - 30/01/2019), assim como pelas demais instituições participantes^14^.

## RESULTADOS

A prevalência geral do uso de múltiplas substâncias foi consideravelmente alta (49,3%). A substância mais frequentemente usada ao longo da vida e nos últimos 12 meses foi o álcool (65,5% em ambos os períodos). Para todas as substâncias, a prevalência de uso nos últimos 12 meses foi menor do que o uso em qualquer momento da vida. O uso de tabaco ao longo da vida foi de 61,6%, comparado com 52,9% nos últimos 12 meses. As taxas de uso de maconha ao longo da vida e nos últimos 12 meses foram de 52,0% e 41,0%, respectivamente. O uso de cocaína ao longo da vida e nos últimos 12 meses foi de 42,6% e 31,0%, respectivamente (Tabela 1).

**Tabela 1:** Distribuição (%) do uso de substâncias ao longo da vida e do uso de substâncias nos últimos 12 meses de acordo com o tipo de substância. São Paulo, Porto Alegre, Salvador, Manaus e Campo Grande, Brasil, 2019–2021.

Substância	Ao longo da vida		12 meses	
	Não	Sim	Não	Sim
	n (%)	n (%)	n (%)	n (%)
Álcool	437 (34,5)	828 (65,5)	437 (34,5)	828 (65,5)
Tabaco	486 (38,4)	781 (61,6)	596 (47,1)	670 (52,9)
Maconha	608 (48,0)	659 (52,0)	759 (59,9)	508 (40,1)
Cocaína	727 (57,4)	540 (42,6)	874 (69,0)	393 (31,0)
Crack	1.091 (86,1)	176 (13,9)	1.172 (92,5)	95 (7,5)
Anfetaminas/ecstasy	1.117 (88,2)	150 (11,8)	1.201 (94,8)	66 (5,2)
Inalantes	1.082 (85,4)	185 (14,6)	1.184 (93,4)	83 (6,6)
Hipnóticos	1.101 (86,9)	166 (13,1)	1.165 (91,9)	102 (8,1)
Alucinógenos	1.138 (89,8)	129 (10,2)	1.202 (94,9)	65 (5,1)
Opioides	1.244 (98,2)	23 (1,8)	1.254 (99,0)	13 (1,0)
Outros	1.250 (98,7)	17 (1,3)	1.257 (99,2)	10 (0,8)

Na Tabela 2 nós mostramos a distribuição dos dados sociodemográficos, engajamento em sexo transacional ao longo da vida, a autoavaliação do estado de saúde, vitimização por violência e o resultado do rastreamento da depressão, estratificado pela presença de uso de múltiplas substâncias. A maioria das entrevistadas tinha 25 anos ou mais (71,3%) de idade, e a grande maioria (74,0%) relatou raça não branca (negra, parda, indígena ou descendente de asiáticos). Uma maioria substancial não estudava no momento da entrevista (78,1%) e 43,1% tinham concluído o ensino fundamental ou menos. A maioria das entrevistadas vivia em condições instáveis de moradia (74,1%), estava envolvida em trabalho informal (79,9%) e tinha o sexo transacional ao longo da vida como sua principal atividade de trabalho (73,7%). Metade das entrevistadas (50,2%) ganhava menos de um salário mínimo brasileiro por mês, 91,9% haviam sofrido algum tipo de violência e 43,9% apresentaram diagnóstico positivo para depressão.

**Tabela 2:** Distribuição (%) e razões de prevalência brutas do uso de múltiplas substâncias de acordo com características sociodemográficas, comportamentos de risco, autoavaliação do estado de saúde, violência e rastreamento para depressão. São Paulo, Porto Alegre, Salvador, Manaus e Campo Grande, Brasil, 2019–2021.

Variáveis	Total (n=1.267)	Uso de múltiplas substâncias^*^		
		Não 643 (50,7)	Sim 624 (49,3)	RP (IC95%)
	n (%)	n (%)	n (%)	
Capitais				
Campo Grande	177 (14,0)	77 (12,0)	100 (16,0)	
Manaus	324 (25,6)	227 (35,3)	97 (15,6)	
Porto Alegre	188 (14,8)	67 (10,4)	121 (19,4)	
Salvador	191 (15,1)	91 (14,2)	100 (16,0)	
São Paulo	387 (30,5)	181 (28,1)	206 (33,0)	
Faixa etária (anos)				
18 a 24	364 (28,7)	160 (44,0)	204 (56,0)	1,27 (1,07–1,51)
25 ou mais	903 (71,3)	483 (53,5)	420 (46,5)	
Raça/cor da pele				
Branco	328 (26,0)	167 (50,9)	161 (49,1)	1,09 (0,90–1,31)
Outros	932 (74,0)	475 (51,0)	457 (49,0)	
Estado civil				
Com parceiro(a)	182 (14,4)	94 (51,6)	88 (48,4)	
Sem parceiro(a)	1.083 (85,6)	549 (50,7)	534 (49,3)	1,05 (0,84–1,32)
Estudo no momento da pesquisa				
Sim	277 (21,9)	135 (48,7)	142 (51,3)	0,96 (0,80–1,16)
Não	988 (78,1)	507 (51,3)	481 (48,7)	
Escolaridade				
Ensino Fundamental ou menos	544 (43,1)	241 (44,3)	303 (55,7)	1,21 (1,04–1,42]
Ensino Médio ou mais	722 (57,0)	401 (55,5)	321 (44,5)	
Moradia				
Casa própria	328 (25,9)	176 (53,7)	152 (46,3)	
Alugada	465 (36,7)	238 (51,2)	227 (48,8)	1,08 (0,88–1,32)
Temporária	474 (37,4)	229 (48,3)	245 (51,7)	1,28 (1,04–1,58)
Trabalho				
Regular	122 (9,7)	81 (66,4)	41 (33,6)	
Instável	1.008 (79,9)	503 (49,9)	505 (50,1)	1,58 (1,14–2,18)
Desempregada	132 (10,5)	56 (42,4)	76 (57,6)	1,68 (1,14–2,46)
Renda mensal				
BRL 0,0–BRL 1,038.00	564 (50,2)	267 (47,3)	297 (52,7)	1,13 (0,96–1,34)
≥BRL 1.038,00	559 (49,8)	285 (51,0)	274 (49,0)	
Sexo transacional ao longo da vida				
Não	333 (26,3)	218 (65,5)	115 (34,5)	
Sim	931 (73,7)	423 (45,4)	508 (54,6)	1,56 (1,27–1,91)
Autoavaliação do estado de saúde				
Muito bom/bom	634 (50,8)	357 (56,3)	277 (43,7)	
Regular/ruim/muito ruim	613 (49,2)	277 (45,2)	336 (54,8)	1,20 (1,02–1,41)
Violência no ano anterior^†^				
Não	103 (8,1)	74 (71,8)	29 (28,2)	
Sim	1.162 (91,9)	568 (48,9)	594 (51,1)	1,79 (1,23–2,60)
Rastreio para depressão				
Positivo	548 (43,9)	245 (44,7)	303 (55,3)	1,17 (1,00–1,38)
Negativo	700 (56,1)	387 (55,3)	313 (44,7)	

*uso múltiplo de álcool e tabaco + uma substância (maconha, cocaína, crack, anfetaminas, inalantes, alucinógenos, sedativos ou opioides);

^†^sofreu pelo menos um tipo de violência: discriminação, agressão verbal, agressão física ou agressão sexual. RP: razão de prevalência; IC: intervalo de confiança.

As características sociodemográficas destacaram o grupo de 18 a 24 anos de idade, o mais comum entre usuários de múltiplas substâncias (56,0%), com chances 1,27 vezes maiores de uso (IC95% 1,07–1,51) em comparação com indivíduos com 25 anos de idade ou mais. Indivíduos com até o ensino fundamental (55,7%) tiveram chances 1,21 maiores (IC95% 1,04–1,42) de se envolverem com o uso de múltiplas substâncias. A moradia temporária foi associada ao uso de múltiplas substâncias (51,7%) em comparação ao não uso (1,28; IC95% 1,04–1,58).

Trabalho esporádico e desemprego foram mais frequentes entre mulheres trans e travestis usuárias de múltiplas substâncias (50,1%; 1,58; IC95% 1,14–2,18 e 57,6%; 1,68; IC95% 1,14–2,46, respectivamente). O engajamento em sexo transacional ao longo da vida foi associado ao uso de múltiplas substâncias (54,6%; 1,56; IC95% 1,27–1,91), assim como pior autoavaliação de saúde (54,8%; 1,20; IC95% 1,02–1,41), relato de situações de violência (51,1%; 1,79; IC95% 1,23–2,60) e rastreamento positivo para depressão (55,3%; 1,17; IC95% 1,00–1,38)).

Na Tabela 3 nós demonstramos o modelo final ajustado de acordo com o menor AIC, no qual as variáveis associadas ao uso concomitante de álcool, tabaco e pelo menos uma substância adicional foram: faixa etária de 18 a 24 anos (1,37; IC95% 1,14–1,65), situação laboral instável (1,52; IC95% 1,08–2,14) ou desemprego (1,58; IC95% 1,05–2,37), sexo transacional ao longo da vida (1,51; IC95% 1,21–1,88) e exposição a alguma violência no ano anterior ao estudo (1,71; IC95% 1,14–2,55).

**Tabela 3:** Análise multivariada de fatores associados ao uso de múltiplas substâncias. São Paulo, Porto Alegre, Salvador, Manaus e Campo Grande, Brasil, 2019–2021.

Variáveis	RPa (IC95%)
Faixa etária (anos)	
18 a 24	1,37 (1,14–1,65)
25 ou mais	1,00
Trabalho	
Regular	1,00
Instável	1,52 (1,08–2,14)
Desempregada	1,58 (1,05–2,37)
Sexo transacional ao longo da vida	
Não	1,00
Sim	1,51 (1,21–1,88)
Violência no ano anterior^*^	
Não	1,00
Sim	1,71 (1,14–2,55)

*sofreu pelo menos um tipo de violência: discriminação, agressão verbal, agressão física ou agressão sexual. Modelo inicial com as variáveis que foram significativas na fase bivariada AIC 1787,7; BIC (*Bayesian Information Criterion*) 1842,5; *Deviance* 1765,7. Modelo final AIC 1785,8; BIC 1820,7; *Deviance* 1771,8. RPa: razão de prevalência ajustada; IC: intervalo de confiança.

## DISCUSSÃO

Neste estudo, realizado em cinco capitais brasileiras, que incluiu 1.317 mulheres trans e travestis, a prevalência do uso de múltiplas substâncias foi de 49,0% e os fatores associados ao uso de múltiplas substâncias foram idade jovem, trabalho instável ou desemprego, sexo transacional e ter sofrido alguma forma de violência.

A população-alvo, na qual os transtornos mentais são frequentes^23^, com alta prevalência de ideação suicida^24^, também se mostrou sujeita a um padrão de uso especialmente intenso e diversificado de várias substâncias psicoativas.

Seria interessante desenvolver análises mais aprofundadas dos riscos e danos associados ao uso simultâneo de álcool e cocaína, amplamente documentados na presente amostra. As duas substâncias são metabolizadas simultaneamente pelo fígado, resultando na produção de cocaetileno, um metabólito altamente tóxico^25^. Esses estudos certamente exigiriam a integração entre o objetivo geral de uma pesquisa comportamental e sorológica e testes toxicológicos, que não foram implementados no contexto deste estudo.

Outro exemplo extremamente relevante envolve os danos e riscos secundários do uso combinado de cigarros e outras substâncias fumadas, inaladas ou vaporizadas (*vaping*), como crack e cigarros eletrônicos. Essas substâncias fumadas e vaporizadas apresentam efeitos sinergicamente adversos nos sistemas respiratório e circulatório e na saúde geral de vários usuários de substâncias^26^. Como tais práticas não são especialmente frequentes e não necessariamente coincidem, a produção de maiores detalhes exigiria uma amostra com esse objetivo específico, fornecendo estimativas com a precisão necessária para explorar o fenômeno em detalhes.

A concentração de taxas mais altas de uso de múltiplas substâncias no subgrupo específico de jovens gera um sinal de alerta, mas também um sinal de esperança. Esse padrão de início do uso de drogas é identificado em contextos e populações amplamente variados, chamando a atenção para a necessidade de intervir preventiva e terapeuticamente o mais cedo possível^27^, combinando abordagens gerais e direcionadas. Um aspecto fundamental é usar instrumentos de rastreamento validados que possam oferecer pistas para profissionais de saúde e para os próprios pacientes como parte de uma rotina abrangente de gerenciamento e cuidado. Uma vez que os sinais precoces se tornem evidentes, cada paciente deve ser avaliado mais detalhadamente, além de oferecer aconselhamento e intervenções breves como uma primeira abordagem^28^.

As usuárias de múltiplas drogas apresentaram maiores chances de não terem vínculo de trabalho formal, o que está relacionado, por sua vez, à baixa escolaridade, de acordo com um padrão complexo de correlação com o uso de substâncias (uma vez que o uso intenso e dependente está frequentemente associado a maiores taxas de abandono escolar). Os resultados de um estudo realizado na Índia, onde as taxas de abandono escolar são muito preocupantes, apontaram fatores semelhantes, destacando o papel das práticas discriminatórias e do uso de substâncias^29^. Vale ressaltar que a comparação de estudos de diferentes culturas usando metodologias e instrumentos distintos requer uma abordagem cautelosa. Em um estudo transversal em que a questão do trabalho e da escolaridade não foi abordada detalhadamente (considerando que esse não era o objetivo), não foi possível estabelecer a direção da associação ou uma possível associação causal entre esses vários fatores.

A educação fornece aos indivíduos os recursos e as habilidades para lidar melhor com os vários desafios da vida. A prevenção do uso de substâncias nocivas nunca é realizada de forma isolada, mas como parte de um contexto de habilidades, conhecimentos e estratégias de enfrentamento bem-sucedidas. Não é por acaso que os programas de prevenção mais bem-sucedidos em todo o mundo não se concentram exclusivamente no uso de substâncias em si, mas em um conjunto de habilidades e capacidades^30^.

As escassas oportunidades de integrar o mercado de trabalho significam que muitas mulheres trans e travestis recorrem ao sexo transacional como uma das poucas (ou mesmo únicas) possibilidades de obtenção de renda e meio de subsistência. O sexo transacional em vários contextos está intimamente associado ao consumo de substâncias por razões muito diversas. Um exemplo são os proprietários de estabelecimentos que incentivam os clientes a consumir bebidas alcoólicas e outras substâncias para aumentar a receita da empresa e a justaposição de redes e vendas de comércio sexual. Além disso, o uso de substâncias é um meio de lidar com o trabalho, que é frequentemente ameaçador e violento (assim redobrando o estigma)^31^.

Para além de qualquer dimensão que afete cada mulheres trans e travestis e suas redes sociais, um contexto geral de transfobia, intolerância e violência certamente contribui para a perpetuação de um círculo vicioso de vulnerabilidade e baixa autoestima. O uso nocivo e dependente de várias substâncias é parte integrante dessa espiral de problemas e sofrimento^11^, que só pode ser mitigada pelo reconhecimento da diversidade do grupo, pela promoção de uma cultura de tolerância e pela eliminação de todas as formas de violência.

Estudos observacionais multicêntricos são, portanto, essenciais, pois evitam vários vieses metodológicos, embora criem outros (como mencionado anteriormente), no impasse que caracteriza dilemas comuns da ciência.

Dois dos vieses metodológicos evitáveis mais relevantes em estudos clínicos envolvendo esse grupo populacional e outras minorias são a autosseleção e a evitação. No primeiro caso, a autosseleção de pacientes, indivíduos mais saudáveis e aqueles que estão mais preocupados com sua própria saúde tendem a demandar mais cuidados clínicos, como pacientes com dependência de álcool com ou sem experiência prévia em grupos de Alcoólicos Anônimos^32^. Intimamente associados e complementando os primeiros estão os pacientes conhecidos por terem hábitos ilegais, com a tendência de evitar interagir com quaisquer serviços formais que exijam inscrição e atendimento regular. A combinação de autosseleção e evitação significa, compreensivelmente, que uma parcela substancial dos usuários de substâncias com uso mais grave simplesmente deixa de interagir com qualquer serviço e acaba sendo identificada exclusivamente por meio de estratégias que não requerem interação humana regular de nenhum tipo, como máquinas de venda automática de seringas^33^.

Referindo-se novamente à análise precisa de Salganik, um viés adicional geralmente conhecido como “viés de desejabilidade social”^11^ tem um forte impacto não apenas em estudos centrados em unidades clínicas, mas também em quaisquer estudos oferecidos a indivíduos que são vistos (ou que se veem) como se comportando de acordo com as normas esperadas de um paciente “bem comportado” e “complacente”. A percepção tem fundamento, uma vez que inúmeros protocolos de intervenção têm critérios rígidos de exclusão, por exemplo, excluindo pacientes com risco de engravidar e indivíduos de vários grupos minoritários, independentemente de tais exclusões serem relevantes^34^.

Portanto, o atual conjunto de estudos pertence a um protocolo idealmente projetado para observar uma série de comportamentos, atitudes e práticas que seriam influenciados inevitavelmente por um protocolo clínico ou intervenção biomédica. Embora o objetivo principal do protocolo original não fosse observar os padrões de uso de substâncias e seus possíveis determinantes, sua implementação se mostrou bastante apropriada para esse fim.

Uma limitação importante do estudo é a inferência estatística para o uso de amostras não probabilísticas. Estudos recentes visam melhorar a inferência, como por exemplo o uso de pseudo-pesos, sem consenso sobre o uso de métodos tão diferentes em estudos utilizando o *respondent-driven sampling.* Diferentes métodos de referência em cadeia foram projetados para explorar as propriedades básicas das redes sociais, avaliando fenômenos definidos como contágios simples, nos quais as cadeias de recrutamento e a difusão de um determinado patógeno (por exemplo, HIV) seguem uma dinâmica semelhante. No entanto, esse não é o caso de comportamentos complexos^35^, e novos métodos de recrutamento de populações de difícil acesso com a avaliação aprofundada do uso de múltiplas substâncias ainda precisam ser implementados^11,35^. Uma segunda limitação diz respeito à ausência de instrumentos desenvolvidos para a avaliação do uso e uso indevido de múltiplas substâncias pela população de mulheres trans e travestis. Uma melhor compreensão dos padrões de difusão de tais comportamentos prejudiciais entre diferentes populações ainda é escassa, e protocolos de intervenção ainda precisam ser desenvolvidos e avaliados.

O Sistema Único de Saúde do Brasil e o setor privado devem incorporar protocolos e procedimentos que atendam às necessidades de diferentes minorias, seguindo os princípios básicos de abrangência e integralidade do cuidado e da gestão. Essa abordagem não deve se limitar ao atendimento especializado, mas ser estendida aos cuidados de saúde primários e comunitários ao menos em seus fundamentos essenciais. Casos complexos devem ser examinados de forma abrangente e encaminhados a serviços especializados, mas a maioria dos pacientes seria melhor tratada e assistida em nível comunitário.

Os profissionais de saúde devem estar cientes e ser devidamente treinados para lidar com condições médicas entre membros de diferentes grupos minoritários, incluindo minorias sexuais e de gênero, bem como indivíduos usuários de múltiplas substâncias. Como princípio orientador, atitudes críticas e preconceitos devem ser evitados.

Nós vivemos em uma sociedade em rápida transição. Os profissionais de saúde devem estar plenamente conscientes da dimensão fundamental da ética e da necessidade premente de oferecer um tratamento humano aos pacientes individualmente, quaisquer que sejam suas identidades sexuais e de gênero e seus hábitos específicos, incluindo o uso de substâncias lícitas e ilícitas.
